# Impacts of Oxazepam on Perch (*Perca fluviatilis*) Behavior: Fish Familiarized to Lake Conditions Do Not Show Predicted Anti-anxiety Response

**DOI:** 10.1021/acs.est.0c05587

**Published:** 2021-03-05

**Authors:** Johan Fahlman, Gustav Hellström, Micael Jonsson, Jerker Berglund Fick, Martin Rosvall, Jonatan Klaminder

**Affiliations:** † Department of Ecology and Environmental Science, Umeå University, Umeå 901 87, Sweden; ‡ Department of Wildlife, Fish, and Environmental Studies, SLU, Umeå 901 83, Sweden; § Department of Chemistry, Umeå University, Umeå 901 87, Sweden; ∥ Department of Physics, Umeå University, Umeå 901 87, Sweden

## Abstract

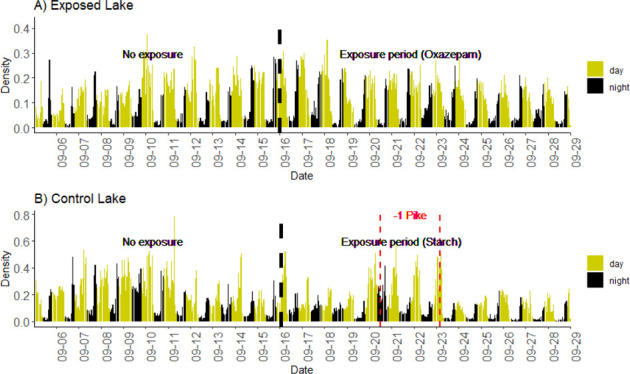

A current theory in environmental science states that dissolved anxiolytics (oxazepam) from wastewater effluents can reduce anti-predator behavior in fish with potentially negative impacts on prey fish populations. Here, we hypothesize that European perch (*Perca fluviatilis*) populations being exposed to oxazepam *in situ* show reduced anti-predator behavior, which has previously been observed for exposed isolated fish in laboratory studies. We tested our hypothesis by exposing a whole-lake ecosystem, containing both perch (prey) and northern pike (*Esox lucius*; predator), to oxazepam while tracking fish behavior before and after exposure in the exposed lake as well as in an unexposed nearby lake (control). Oxazepam concentrations in the exposed lake ranged between 11 and 24 μg L^–1^, which is >200 times higher than concentrations reported for European rivers. In contrast to our hypothesis, we did not observe an oxazepam-induced reduction in anti-predator behavior, inferred from perch swimming activity, distance to predators, distance to conspecifics, home-range size, and habitat use. In fact, exposure to oxazepam instead stimulated anti-predator behavior (decreased activity, decreased distance to conspecifics, and increased littoral habitat use) when using behavior in the control lake as a reference. Shoal dynamics and temperature changes may have masked modest reductions in anti-predator behavior due to oxazepam. Although we cannot fully resolve the mechanism(s) behind our observations, our results indicate that the effects of oxazepam on perch behavior in a familiar natural ecosystem are negligible in comparison to the effects of other environmental conditions.

## Introduction

Pharmaceuticals are ubiquitous in freshwater ecosystems,^1^ and their ability to propagate through freshwater^2^ and riparian food webs^3^ creates a concern for their environmental impact.^4^ Here, collapsing fish populations, due to additions of synthetic estrogen and their direct effect on fish reproduction, serve as one striking example of how pharmaceuticals can impact aquatic ecosystem.^5^ A less direct environmental risk comes from dissolved anxiolytic drugs that reduce anti-predator behaviors and, thus, threaten to increase mortality for prey fish^6^ or change ecosystem structure.^7^ However, researchers currently struggle to show that non-lethal effects, such as behavioral modifications, from chemical stressors are expressed in natural aquatic ecosystems where drugs are greatly diluted.^8^ Hence, there is an urgent need to test whether or not the non-lethal effects from pharmaceutical exposure observed in laboratories are present also in complex, natural environments.

Oxazepam is an anxiolytic in the benzodiazepine family that lowers the action potential of the GABAa receptor in the central nervous system, bioaccumulates in fish,^2^ and persists for months in freshwater,^9^ or even for decades in sediments.^10^ Furthermore, oxazepam has well-documented effects on fish anti-predator behavior in laboratory-based trials^11,12^ and when pre-exposed fish are released into natural ecosystems.^13−15^ For example, laboratory trials have shown that oxazepam causes bolder and more risk-taking behavior in European perch (*Perca fluviatilis*), that is, increased swimming activity, increased willingness to explore new areas, and reduced social interactions with conspecifics.^6,11,12,14,16^ When pre-exposed individuals were released into a natural lake, the initial behavioral responses were as predicted based on the laboratory trials, that is, increased swimming activity, larger home range, reduced social interactions, and a preference for more risky habitats.^14^ Ecological theory predicts that if fish express this bold and active behavior in nature, they will become more exposed to predators and thereby experience increased mortality.^17^ Indeed, a study that released oxazepam-exposed Atlantic salmon (*Salmo salar*) smolt into a river confirms that exposed fish initially can experience increased predation.^15^ However, a more long-term (70 days) field study, exposing European perch and northern pike (*Esox lucius*) to oxazepam in pond ecosystems, did not find any significant predation effects,^18^ for reasons that remain unresolved. However, no study till date have assessed oxazepam-induced behavioral responses in fish being contaminated within a natural system, where fish are not stressed by human handling and exposure to a novel environment—treatments that have been implicit in previous studies finding effect of oxazepam on fish behavior.

Oxazepam may fail to affect predation if prey fish develop tolerance to the drug over time,^19^ or if individual fish responds differently to oxazepam exposure when in groups (shoals, in natural systems) than when in isolation (small aquaria, in most laboratory studies); a notion that finds support in ecological theory on collective decision-making^20^ and in studies finding no behavioral effects of oxazepam when exposed fish enters risk areas as a group.^21^ Similarly, a pioneering study found that effects of a psychoactive substance on fish behavior depended on interactions with conspecifics as effects were only expressed when measured on the shoal level and not on the individual level.^22^ Furthermore, in natural environments, numerous conditions may influence fish behavior, and a recent study found that water temperature may generate larger behavioral responses than oxazepam.^16^ Hence, variation in natural conditions, such as water temperature and light, may moderate behavioral and predation effects caused by oxazepam in natural systems.

Behavioral modifications in fish exposed to oxazepam have been observed in laboratory trials and field studies at concentrations of 0.84–2 μg L^–1^.^12,13,23^ As a comparison, oxazepam concentration in European rivers is typically below 0.061 μg L^-1,^,^24^ and the most contaminated system to our knowledge (River Fyris, Sweden) shows concentrations ranging between 0.21 and 0.58 μg L^–1^.^10,11^ In this study, we assess whether exposure to oxazepam at a concentration (>11 μg oxazepam L^–1^) well above those known to alter perch behavior, and those found in contaminated systems, can also alter *in situ* behavior in perch habituated to a natural lake environment. We conducted a study in which oxazepam was added to a whole lake while fish behaviors were measured over the long term, both before and after the oxazepam addition. We hypothesized that the oxazepam contamination would, at least initially, cause a significant increase in swimming activity, exploratory behavior (increased home-range), and reduced predator avoidance. However, we also monitored shoaling and water temperature, to be able to relate the importance of possible oxazepam-induced behavioral effects to other caused by external factors in the surrounding environment.

## Materials and Methods

### Catching and Tagging

The methods are derived from the field study described in detail by Fahlman *et al.* (2020).^40^ In July 2016, we caught Eurasian perch (size 166 ± 27 mm) in Lake Stöcksjön (63°45′45.9″N 20°11′54.1″E) close to Umeå in northern Sweden, using a beach seine net. In this lake, northern pike is a key predator, assuring that the perch was adapted to cues from this predator. We transported the perch in 1 m^3^ oxygenated flow-through tanks at 15 ± 1.5 °C to Umeå Marine Research Facility (UMF). Shortly after, we anesthetized 44 perches using MS-222 and surgically inserted acoustic transmitters (VEMCO V4 180 kHz, 4.3 × 12.7 mm, 0.64 g) into the abdominal cavity. We closed the incision with a suture and left the fish to recover for a minimum of 14 days before any further handling. Every individual was eating chironomid larvae during the tagging and recovery phase of the study and appeared healthy by the end of the recovery time.

### Field Site

The field site consisted of two twin lakes close to Åmsele, Västerbotten, Sweden (64°29′2.5″N 19°25′8.2″E). Both lakes (here named Exposed Lake and Control Lake) are kettle-hole lakes, each with an area of about 4000 m^2^, a maximum depth of roughly 6 m, and a border of quagmire covering the shoreline. The lakes and their food resources are described in detail in ref (25). Measured with HOBO loggers and point measurements, the water temperature was 12 ± 1.5 °C (Figure S1) during the study. To calculate daily water temperatures between point measurements, we used a best-fit function (polynomial regression, *R*^2^ = 0.51) between air temperatures and measured water temperatures (*n* = 64). The lakes were fishless before the experiment due to rotenone extermination of the natural perch populations in the lakes during 1995.^26^ Resource sampling showed an abundance of pelagic (*Daphnia* zooplankton and *Chaoborus* larvae) and benthic (mainly *Ephemeoptera, Odonata*, and *Asellus aquaticus*) invertebrates. One day before we introduced the perch, we stocked each lake with four northern pikes (*E. lucius*; length 600 ± 50 mm), caught in Lake Tavelsjön (64°0′2.4″N 20°3′5.1″E) and tagged in the same manner as described above for the perch. This pike density is within the lower range of what has been previously shown in nearby lakes^27^ and generate a perch/pike ratio around 5, which is somewhat lower than that in the lake where the perch originated that has a reported ratio of 15–22.^28^ However, the experimental set-up largely resembles that of a previous oxazepam experiment with pre-exposed pike (*n* = 4) and perch (*n* = 34).^14^ Given the relatively low densities of prey (∼55 ha^–1^), the pikes’ main purpose was not to measure predation impact but instead to induce chemical and visual predator cues and thus create a landscape of perceived risk. This “landscape of fear” is known to induce animals’ natural behavior in the wild.^29^ We tracked the fish in the lakes using the VEMCO HR2 system,^30^ with eight receivers attached to anchored buoy lines in each lake. Three additional VEMCO VR2w receivers in the Control Lake covered a small bay behind an island. This setup ensured complete coverage of the lakes. If three or more receivers could detect a tagged fish simultaneously, its position could be triangulated using hyperbolic positioning methods.^31^ We tracked the fish for 25 days (the transmission interval was on average 2s) and evaluated the positional accuracy using horizontal positioning error and root-mean-square error.^31^

### Fish Release and Oxazepam Exposure

**Figure 1:**
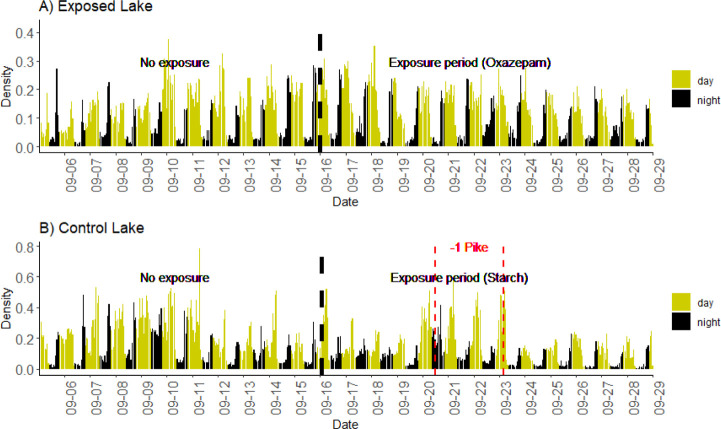
Diurnal cycle in shoaling behavior for perch. Social network density during the study period in (a) the Exposed Lake treated with oxazepam and (b) the Control Lake. Yellow bars indicate hourly measures during the day, and black bars indicate nighttime measures. Analytical data are based on daily means. The daily changes in social associations occur before the sunrise and the sunset; the fish show asocial behavior during the night. A vertical dashed black line shows the time of spiking, while the vertical dashed red line indicates periods where the Control Lake experienced pike mortality.

After the tagging and recovery procedure, on September 5, we transported the perch to the field site in oxygenated tanks and randomly distributed them between the two lakes, with 22 perch introduced to each lake. We left the fish undisturbed for 11 days until September 16, when we exposed the Exposed Lake to oxazepam (Oxascand TEVA), with a nominal concentration goal of 15 μg L^–1^. This concentration is well above the range 0.57–2.1 μg L^–1^, where oxazepam has been shown to affect fish behavior^11,12,32^—thereby avoiding a situation where too low exposure in the lake would make comparisons between previous documented behavioral responses difficult. We manually poured about 150 L of dissolved oxazepam from plastic cans into the Exposed Lake (left in Figure 1) while traversing the lake in transects in a boat. The Control Lake (right in Figure 1) received the same treatment, but with starch placebos, mimicking the matrix in the used Oxascand pills, at the same quantity as the Dosed Lake. We sampled oxazepam twice per week at every meter on nine different sites (*n* = 38) scattered across the exposed Exposed Lake and at two sites in the Control Lake. Measured oxazepam concentrations (measured by LC–MS/MS and detailed description of the analytical methods are found in ref (9)) were 24.1 ± 9.7 μg L^–1^ (±1 SD) shortly after exposure and 11.4 ± 1.5 μg L^–1^ at the end of the study period (Table S1). Average during the whole study (sampling period: September 20 to September 30) was 14.9 ± 7.4 μg L^–1^. On September 21, we observed predation of a pike by a white-tailed eagle. Therefore, we restocked with a new pike (September 23), which caused the Control Lake to function with one less predator between these dates. We terminated the experiment on September 29, when the acoustic transmitter batteries reached end of life.

### Data Analysis

To ensure consistency and comparability over time, we interpolated fish positioning data for every 60 s, thereby avoiding differing number of data points for a given time period. From these data, we extracted four field variables for perch: (1) activity (swimming speed in m minute^–1^); (2) pelagic use (distance to the shoreline in meter); (3) home range [95% of the used area, calculated as the mean convex polygon (MCP) in m^2^]; (4) predator avoidance [distance to the closest predator (*i.e.*, pike) in meter]; and (5) social distance (distance to the closest conspecific in meter). We also measured the first three traits for pike. Note that previous studies have suggested that these measurements are suitable field analogues for detecting oxazepam-induced behavioral modifications.^14^

We determined the activity by measuring the distance traveled between interpolated data points every 60 s and the pelagic use by measuring the shortest distance to the shoreline every 60 s. Extracted as a daily measurement, we calculated the home range as the 95% MCP. The total number of interpolated data points (*i.e.*, positions) was about 1,900,000. For analytical purposes and to account for circadian rhythms in behavior, we calculated two population averages per day of these measurements; one for daytime and one for nighttime.

Previous studies have shown that social interactions are essential for individual fish decision making.^33,34^ To be able to assess potential impact of the collective on individual fish behavior, we considered the social network structure as a possible external factor affecting individual behavior. In short, we quantified the social network density, the ratio between the number of existing connections, and the number of possible connections^35^ at the time of each behavioral measure. Note that this measure is more strongly dependent on the activity of the collective than the distance to the nearest neighbor, which is a measure previously used as a proxy for social behavior of individuals.^14^ Because an individual’s ability to affect the behavior of all fishes in the lake is low, the use of social network density as an external driver of behavior is reasonable. Using the “spatsoc” package for R, and following the methods in ref (36), we extracted point-based spatial groupings with a distance threshold of 1 m (precision limit of the acoustic telemetry system) for social connections from the minute-interpolated data and calculated the population averages of every day and night.

### Statistical Methods

When testing for the effects of oxazepam exposure on the selected traits, we used multiple linear regression models (LMs) containing the predictors time, treatment, and the measured environmental factors, that is, lm(trait ∼ time × treatment × temperature × social network density). Analysis was divided into two time steps: the first 0–48 h and 2–25 days after oxazepam exposure. The rationale for this was to: (i) improve our ability to detect an eventual initial short-term behavioral response that diminished over time and (ii) assure that the analysis conducted on the 2–25 day data was based on fish that had experienced enough time for a significant uptake of the drug. We analyzed measured behavioral traits using a before-after-control-impact (BACI) approach, with data divided into time (before [B] and after [A] oxazepam addition) and treatment (control [C] and treatment [I]). We used the interaction time × treatment as a criterion for rejecting the null hypothesis that oxazepam does not affect a given behavioral trait in perch. In the analysis, night and day behavior was modeled separately as previous whole-genome microarray analyses have indicated that benzodiazepines may activate fish genes involved in the circadian rhythm.^37^ For the period 0–48 h, hourly means for each fish were used while two daily population averages (day and night) per trait were used for the period 2–25 days after exposure. To find the most parsimonious models, we performed stepwise AIC model selection,^38^ using the “MASS” package for R. Visual inspection of residuals confirmed normally distributed data. We used the statistical software R (version 3.6.3^39^) for all analyses.

## Results

### System Performance

The receivers successfully detected all tagged fish over the entire study period. Positional accuracy remained below 1 m for most part of the lakes, with performance data presented in ref (40). The overall detection rate was on average 50% for both lakes, sufficient for getting perch and pike positions interpolated at 1 min resolution. Pikes in the Control Lake generated on average 25% more interpolated data points per hour than the pikes in the Exposed Lake. This discrepancy was likely due to the pikes’ tendency to remain around a small island that blocked some signals and in vegetated areas with poorer acoustic conditions in this latter lake. Lower signal detections for pike in the Exposed Lake caused systematically longer distances to the nearest predator. Hence, to avoid measuring effects simply caused by fewer interpolated data points, we reduced this bias by normalizing the calculated distance to the nearest predator to the between-lake differences in the signal frequency (*i.e.*, distance to a predator × relative difference in signals between the two lakes). Figure S2A–D illustrates the perch positioning data in the two lakes and the conversion of positions into social networks that capture the shoaling behavior. The shoaling density in the two lakes was highest during the daytime, with sharp increases and decreases at dawn and dusk, respectively, creating diurnal cycles in shoaling densities (Figure 1A,B).

### Behavioral Effects Caused by *In Situ* Oxazepam Exposure

**Figure 2:**
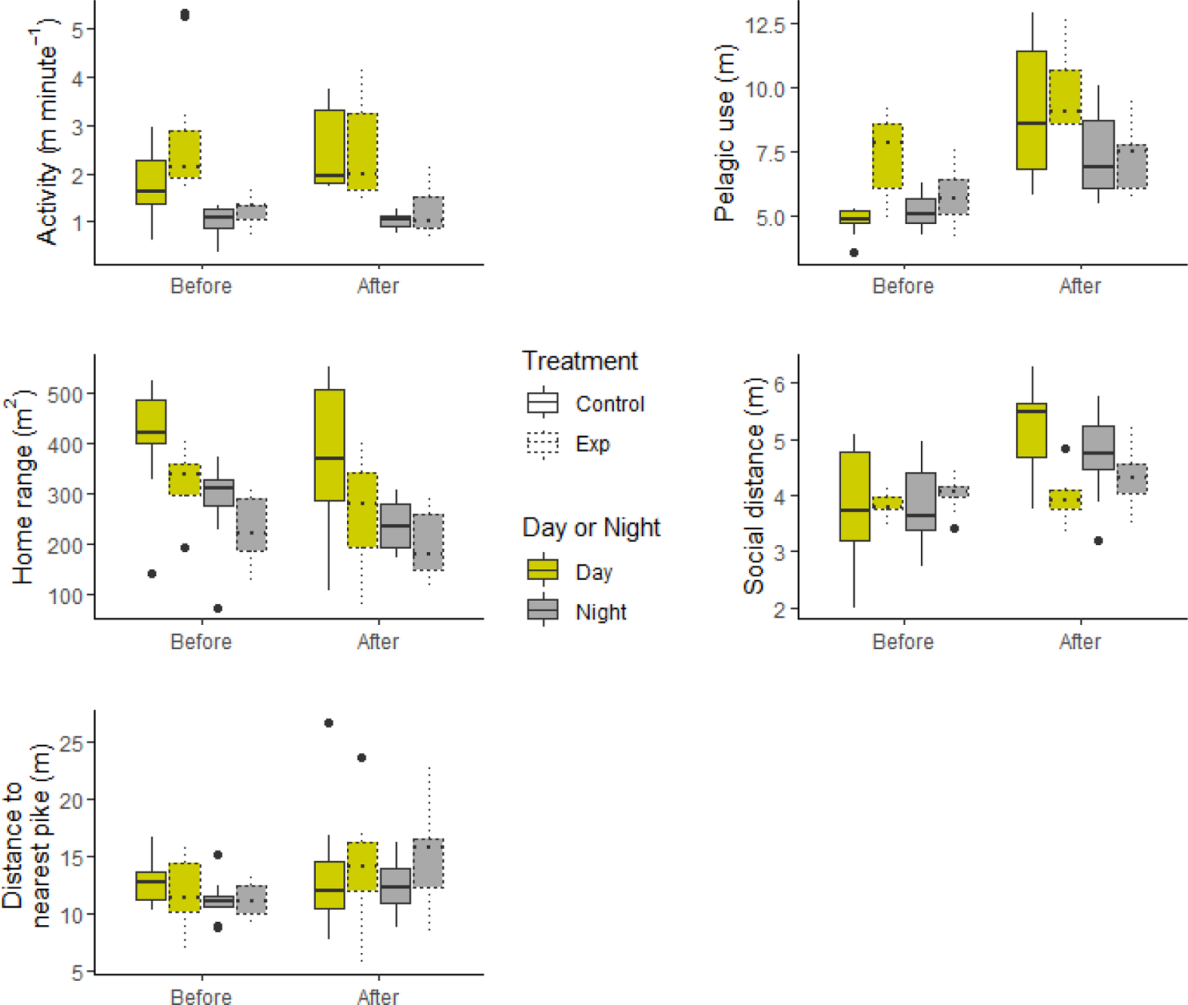
Oxazepam treatment effect on perch behavior. Boxplots displaying the effect of oxazepam treatment on the four measured behavioral traits. Solid boxes are measurements in the Control Lake and dotted boxes in the Exposed Lake. Yellow boxes represent daytime measurements, and gray boxes represent nighttime measurements.

There were no significant effects of oxazepam, that is, a significant time × treatment interaction (hereafter called “oxazepam effect”), on pike behavior (Figure S3). During the first two days after exposure, the oxazepam had no effect on any of the measured traits for perch (Table S1). During day 2–25, oxazepam exposure decreased perch activity during day and night (*p* = 0.002–0.014), as well as increased littoral use (*p* = 0.013) and decreased social distance during daytime (*p* = 0.006) (Table 1; Figure 2). Interestingly, in several cases, the oxazepam effect interacted with temperature or shoal density to influence perch behaviors. For example, the effect of oxazepam on daytime pelagic use was reduced by increasing shoal density (*p* = 0.002) and seemed enhanced by the increasing temperature (*p* = 0.007) (Table 1).

**Table 1:** Summary of Results from Multivariate LMs^a^

Day															
	pelagic use			home range			activity			predator avoidance			social distance		
predictor	estimate	CI	*p*	estimate	CI	*p*	estimate	CI	*p*	estimate	CI	*p*	estimate	CI	*p*
(intercept)	94.53	62.71 to 126.34	**<0.001**	–3575.32	–5953.88 to −1196.76	**0.004**	11.71	–7.50 to 30.92	0.225	30.89	3.05 to 58.72	**0.03**	6.47	5.86 to 7.08	**<0.001**
time (before)	–78.97	–132.37 to −25.57	**0.005**	4651.34	591.84 to 8710.84	**0.026**	–1.05	–1.78 to −0.32	**0.006**				–1.01	–1.48 to −0.54	**<0.001**
treatment (exposed)	–35.87	–52.09 to −19.64	**<0.001**	1054.76	–159.36 to 2268.87	0.086	–11.11	–21.33 to −0.88	**0.034**	–31.56	–70.93 to 7.80	0.113	–2.52	–3.65 to −1.38	**<0.001**
density	–253.8	–417.28 to −90.32	**0.003**	12,757.69	1128.15 to 24,387.24	**0.033**	–44.53	–26.89 to 37.83	0.281				–6.94	–9.89 to −4.00	**<0.001**
temperature	–7.14	–9.79 to −4.49	**<0.001**	318.59	119.12 to 518.06	**0.003**	–0.93	–2.57 to 0.71	0.259	–1.5	–3.82 to 0.83	0.2			
*time (before) × treatment (exposed)*	*34.46*	*7.75 to 61.18*	***0.013***	*–1482.38*	*–3497.52 to 532.77*	*0.144*	*1.52*	*0.59 to 2.45*	***0.002***				*0.93*	*0.29 to 1.57*	***0.006***
time (before) × density	210.7	–31.90 to 453.29	0.086	–15,406.47	33,417.35 to 2604.42	0.091									
treatment (exposed) × density	–20.41	–47.38 to 6.56	0.133										6.69	–0.31 to 13.69	0.06
time (before) × temperature	6.17	1.61 to 10.73	**0.01**	–388.85	–735.84 to −41.85	**0.029**									
treatment (exposed) × temperature	3.32	1.85 to 4.79	**<0.001**	–96.04	–198.19 to 6.11	0.064	0.96	0.10–1.82	**0.03**	2.65	–0.64 to 5.94	0.111			
density × temperature	20.92	7.85 to 34.00	**0.003**	–989.14	–1928.71 to −49.58	**0.04**	4.48	–2.40 to 11.36	0.195						
[time (before) × treatment (exposed)] × density	52.62	20.84 to 84.41	**0.002**												
[time (before) × treatment (exposed)] × temperature	–3.38	–5.77 to −1.00	**0.007**	130.2	–41.55 to 301.94	0.133									
[time (before) × density] × temperature	–17.03	–37.16 to 3.09	0.094	1272.01	–231.81 to 2775.83	0.095									
observations	46			46			46			46			46		
*R*^2^/*R*_adjusted_^2^	0.913/0.878			0.750/0.669			0.588/0.512			0.061/–0.007			0.677/0.637		

Night															
	pelagic use			home range			activity			predator avoidance			social distance		
predictor	estimate	CI	*p*	estimate	CI	*p*	estimate	CI	p	estimate	CI	*p*	estimate	CI	*p*
(intercept)	50.08	25.21 to 74.94	**<0.001**	–511.58	–902.49 to −120.67	**0.012**	–2.84	–8.90 to 3.23	0.348	36.35	18.26 to 54.45	**<0.001**	20.24	7.18 to 33.30	**0.004**
time (before)	–49.55	–117.50 to −18.40	0.147	712.11	87.58–1336.64	**0.027**	–2.21	–7.18 to 2.75	0.371	–2.63	–4.12 to −1.14	**0.001**	–18.1	–53.80 to 17.59	0.309
treatment (exposed)	–74.14	–129.15 to −19.13	**0.01**	–50.8	–246.22 to 144.61	0.601	–5.54	–9.55 to −1.52	**0.008**	–32.58	–57.72 to −7.43	**0.012**	–36.37	–65.27 to −7.48	**0.015**
density	–231.02	–498.57 to 36.53	0.088	–25.21	–1019.57 to 969.15	0.959	44.72	–7.54 to 106.99	0.153				–82.12	–222.65 to 58.42	0.242
temperature	–3.53	–5.59 to −1.46	**0.002**	62.18	27.66–96.70	**0.001**	0.3	–0.21 to 0.81	0.238	–1.95	–3.45 to −0.45	**0.012**	–1.25	–2.34 to −0.17	**0.025**
*time (before) × treatment (exposed)*	*91.03*	*–5.58 to 187.65*	*0.064*	*196.68*	*–73.39 to 466.75*	*0.148*	*8.33*	*1.82 to 14.83*	***0.014***				*42.08*	*–8.67 to 92.82*	*0.101*
time (before) × density	268.72	–293.00 to 830.45	0.336	676.81	–523.24 to 1876.86	0.26	–6.34	–13.86 to 1.18	0.096				105.64	–89.41 to 400.69	0.47
treatment (exposed) × density	759.34	73.74 to 1444.94	**0.031**	124.21	–2392.67 to 2641.09	0.921	10.23	–3.00 to 23.45	0.125				277.2	–82.92 to 637.32	0.126
time (before) × temperature	4.01	–1.81 to 9.82	0.17	–62.15	–116.40 to −7.89	**0.026**	0.23	–0.21 to 0.68	0.293				1.46	–1.60 to 4.51	0.338
treatment (exposed) × temperature	6.1	1.49 to 10.70	**0.011**				0.41	0.06 to 0.75	**0.024**	2.83	0.73 to 4.93	**0.01**	3.05	0.64 to 5.47	**0.015**
density × temperature	18.73	–2.66 to 40.12	0.084				–3.38	–8.35 to 1.59	0.176				6.27	–4.96 to 17.51	0.263
[time (before) × treatment (exposed)] × density	–959.24	–1998.88 to 80.40	0.069	–2273.29	–5632.36 to 1085.77	0.178	–12.2	–29.66 to 5.26	0.164				–373.88	919.96 to 172.20	0.172
[time (before) × treatment (exposed)] × temperature	–7.82	–15.99 to 0.36	0.06				–0.62	–1.19 to −0.05	**0.033**				–3.58	–7.87 to 0.72	0.099
[time (before) × density] × temperature	–22.52	–69.83 to 24.78	0.339										–8.75	–33.60 to 16.10	0.478
[treatment (exposed) × density] × temperature	–62.14	–119.03 to −5.25	**0.033**										–23.8	–53.68 to 6.08	0.114
[time (before) × treatment (exposed)] × density × temperature	83.07	–3.76 to 169.90	0.06										32.36	–13.25 to 77.97	0.158
observations	46			46			46			46			46		
*R*^2^/*R*_adjusted_^2^	0.759/0.638			0.516/0.394			0.557/0.396			0.359/0.296			0.674/0.511		

a The effect of oxazepam on the measured behavioral traits is tested by the time × treatment interaction (row set in italics) according to the BACI design. The best-fitting models selected using stepwise AIC model selection are shown.

### Behaviors Affected by Environmental Factors

**Figure 3:**
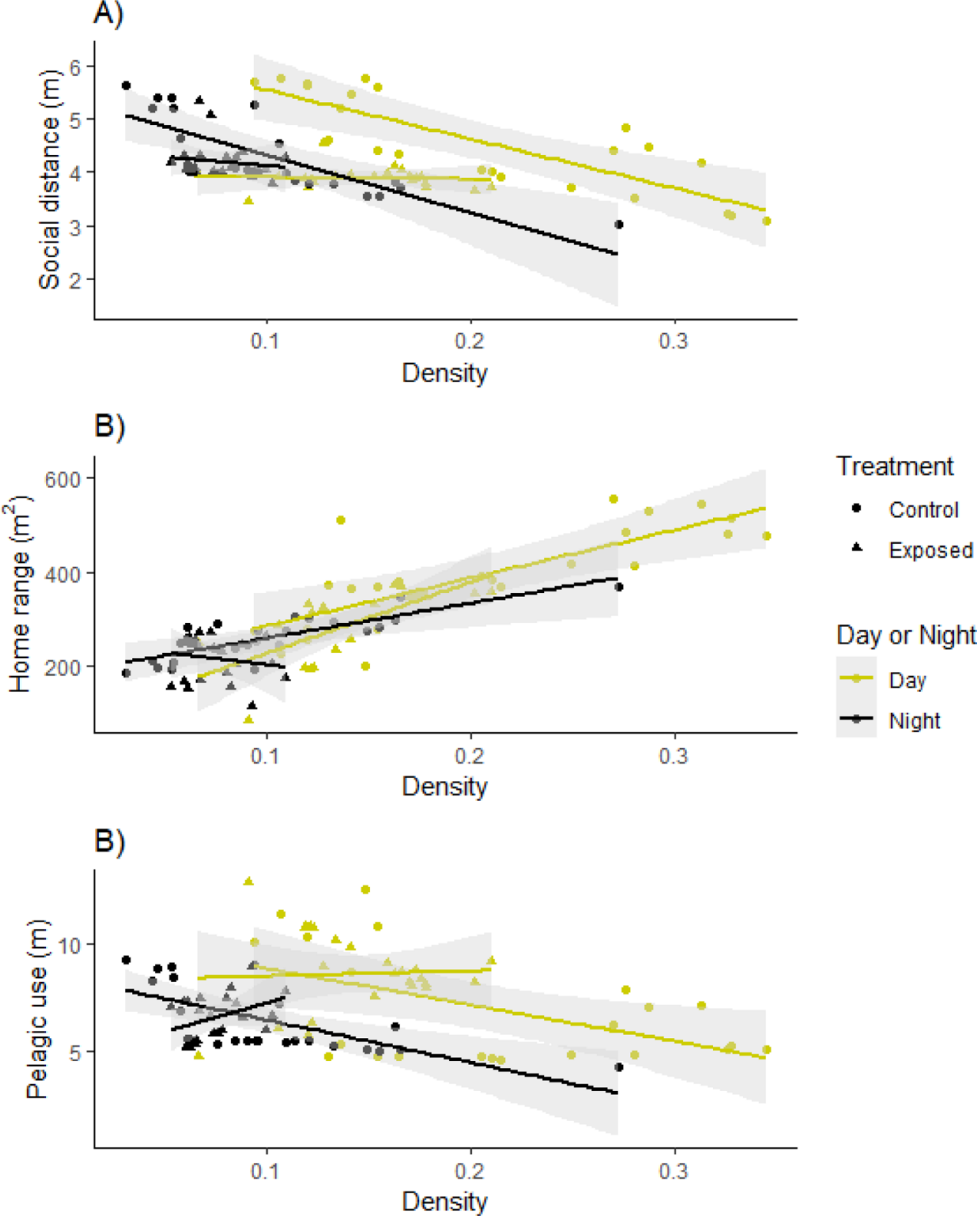
Effect of social network density on the measured behavioral traits in both studied lakes. Predicted values of social network density derived from the multivariate LMs in relation to (a) social distance, (b) home-range size, and (c) pelagic use. Data are from daily averages of the respective populations (Control Lake and Exposed Lake). Linear model trends, shown as lines and shaded areas, represent 95% confidence intervals. Note that of all trends shown, only daytime values are significant (*p* < 0.05).

Social network density was an influential factor in controlling several measured behavioral traits (Table 1; Figure 3). A well-connected social network, which corresponds to a high shoaling density score, was positively related to inferred high-risk behavior, such as a higher level of pelagic use and larger home range (Figure 3). Water temperature was the dominant factor that affected habitat selection and also had an impact on home-range size and social distance (Table 1). The small changes in water temperature (Δ = 2.5 °C) observed during the study period had a significant effect: higher temperature caused the perch to increase their use of littoral habitats while limiting total home range during both night and day. At nighttime, social distance and predator avoidance decreased as an effect of higher temperature. The effect of shoal density was temperature dependent (Table 1). For example, the negative effect of social network density on pelagic use decreased with increasing temperature. Similarly, the positive effect of social network density on home range size decreased with increasing temperature (Table 1).

## Discussion

Numerous studies have found that oxazepam contamination can reduce perch anti-predator behavior with potential increases in predation as a consequence.^6,11,12,16^ In contrast to these findings and our hypothesis, we found that the studied anxiolytic did not reduce perch anti-predator behavior. Instead, oxazepam had no effect, or even increased anti-predator behavior (*i.e.*, reduced activity and decreased daytime pelagic use). These findings, that oxazepam did not reduce anti-predator behavior at exposures around 14.9 ± 7.4 oxazepam μg L^–1^, are supported by a previous long-term study that found no oxazepam-induced predation on perch by pike in ponds contaminated with 15.5 ± 4 oxazepam μg L^–1^.^18^ Fish developing tolerance to the drug can be ruled out as an explanation to the absence of expected effect in our study as that would generate an initial response that diminished over a time scale of weeks,^32^ which was not the case in this study (Table S2). Instead, we observed for the full period of 2–25 days after exposure that oxazepam reduced perch activity as well as increased use of the littoral zone at daytime, which is commonly interpreted as increased anti-predator behavior.^14^ Nevertheless, our results, along with those of Lagesson *et al.*,^18^ suggest that oxazepam concentrations two magnitudes higher than that typically found in contaminated rivers^41^ seem unlikely to increase the vulnerability of perches to predators.

Our exposure concentrations were intermediate to those used in previous studies where fish has been exposed to oxazepam in laboratory settings and released into natural ecosystems. That is, exposure concentrations in these previous studies range from around 1.9 μg oxazepam L^–1^^13^ to 200 μg oxazepam L^–1^.^14,15^ Effects of oxazepam are likely to be dose dependent, but a dose-dependent response cannot explain why fish at intermediate concentrations would show a response completely opposite to that expected. We cannot fully resolve the mechanism(s) behind the rejection of our hypothesis, but we list three causes that may have affected the result: (i) “oxazepam effect” might be negligible in comparison to effects of other environmental factors. For example, the 2.5 °C variation in water temperature during the study period more than doubled the perch’s nighttime activity and increased their overall littoral use by 25%, which may have masked weak responses to oxazepam exposure. Nevertheless, effects of oxazepam may have been noticeable if temperature would have been constant. (ii) The effect of oxazepam is context dependent. That the effect of oxazepam is dependent on the specific environment is evident from the significant interactions between oxazepam effects and temperature or shoaling density. One major difference between our study and previous studies observing a reduction in anti-predator behavior caused by oxazepam^6,11−14,16,19,21,23,32,42^ is that our perch was not recently subjected to artificial stress, such as isolation and human handling, or exposed to a novel environment during the behavioral trial. The theory that artificial treatments may affect perch response to oxazepam is supported by studies showing that stress from isolation in combination with human handling increases the uptake of the drug.^43^ In line with the notion that oxazepam may generate stronger effects in an unfamiliar environment is a study that showed that wild-caught zebrafish (*Danio rerio*), not adapted to the laboratory environment, was reducing its anti-predator behavior in response to oxazepam, while laboratory strains adapted to laboratory environments showed no response.^32^ The importance of the novel environment for the oxazepam effect is also indicated by a study showing that *D. rerio* becomes unresponsive to oxazepam when being reintroduced into a familiar environment,^19^ but this could also be due to fish developing a physical tolerance to the drug, as interpreted by the authors. (iii) The oxazepam effect on the collective (shoal) differs from that measured on single individuals. Collective decision-making generates robustness against individuals’ erratic behaviors, as suggested by modeling efforts,^20^ and it seems possible that formation of shoals within the lake may have made behavior of individual fishes more resistant to the drug. Indeed, shoaling seem to be part of most of the risky behaviors measured on our study. The importance of the shoal for individual fish behavior highlights the conceptual problem of using behavioral tests on isolated individuals (in the laboratory) for predicting how fish will act in nature, where interactions with conspecifics strongly influence anti-predator behaviors.

From a historical perspective, whole-lake experiments have been crucial for demonstrating the impact of a stressor on aquatic ecosystems. For example, hallmark studies outlining impacts of eutrophication,^44^ acidification,^45^ synthetic estrogen,^46^ and early warning signs for regime shifts,^47^ were all conducted on the whole-lake scale to provide realism to previous laboratory experiments. Our whole-lake study indicates that oxazepam does not generate a detectable reduction in anti-predator behavior in nature, even at concentrations above what has ever been measured in contemporary aquatic ecosystems. This finding, in combination with that of a previous pond experiment showing no long-term effects of oxazepam on perch survival and growth rates,^18^ suggests that oxazepam contamination likely poses a less potent threat to aquatic ecosystems than what is currently believed.

## Supporting Information Available

Water temperature data for the study lakes, visualization of perch shoaling behavior analysis, pike activity, pelagic use and home range data, oxazepam sampling data from the study lakes, and statistical modeling of behavioral traits for perch (PDF)

## Notes

This research was approved by the Ethical Committee on Animal Experiments in Umeå (dnr: A56-14) and complied with Swedish law.
