# Supplementary material for: Impacts of Oxazepam on Perch (Perca fluviatilis) Behavior: Fish Familiarized to Lake Conditions Do Not Show Predicted Anti-anxiety Response
Source: Environ Sci Technol. 2021 Mar 5;55(6):3624–33. doi: 10.1021/acs.est.0c05587 (PMC8031365; doi:10.1021/acs.est.0c05587)
Supplement: Supplementary file 1 — es0c05587_si_001.pdf [file es0c05587_si_001.pdf]

Impacts of oxazepam on Perch (*Perca fluviatilis*) behavior: fish familiarized to lake conditions do not show predicted anti-anxiety response: Supporting information

Authors: Johan Fahlman[1]*, Gustav Hellström[2], Micael Jonsson[1], Jerker Berglund Fick[3], Martin Rosvall[4], Jonatan Klaminder[1]

Affiliations:

[1]Department of Ecology and Environmental Science, Umeå University, Umeå, Sweden.

[2]Department of Wildlife, Fish, and Environmental Studies, SLU, Umeå, Sweden.

[3]Department of Chemistry, Umeå University, Umeå, Sweden.

[4]Department of Physics, Umeå University, Umeå, Sweden.

*Correspondence to: johan.fahlman@umu.se

Contents:

Page 1:

Fig S1: Water temperature data from the study lakes

Page 2:

Fig S2: Visual representation of perch shoaling behavior

Page 3:

Fig S3: Oxazepam effect on pike behavior

Page 4:

Table S1: Oxazepam sampling data

Page 5:

Table S2: Summary of statistical modelling on perch behavior

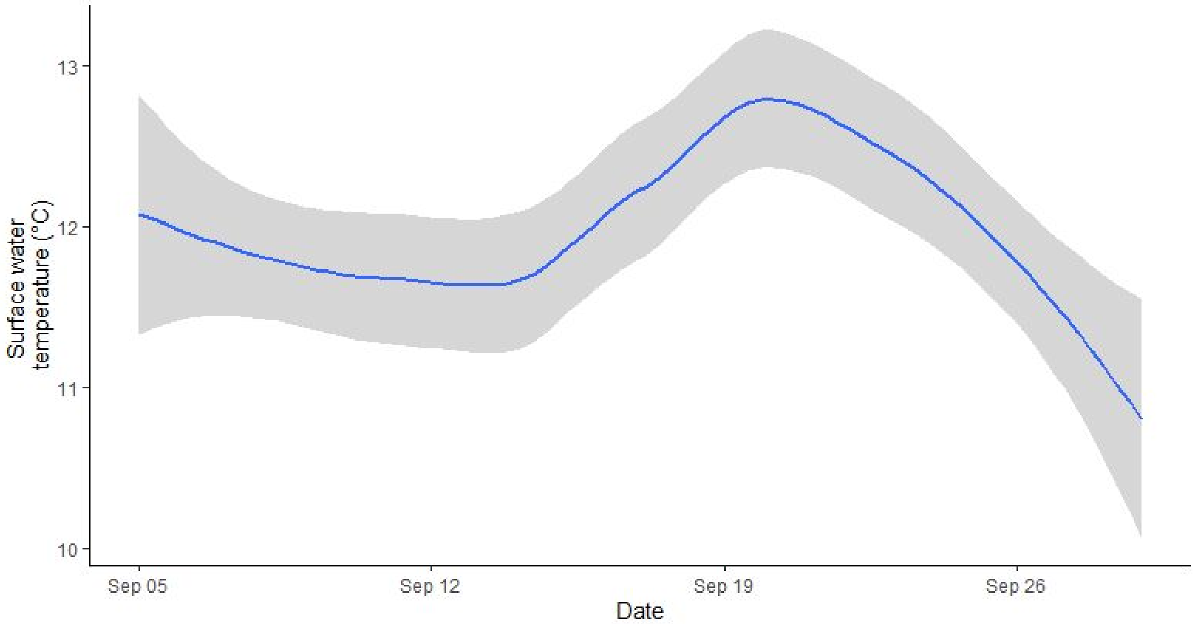

Fig. S1. **Surface water temperature.** The surface water temperature (at 0.5 m depth) of the lakes during the whole study period.

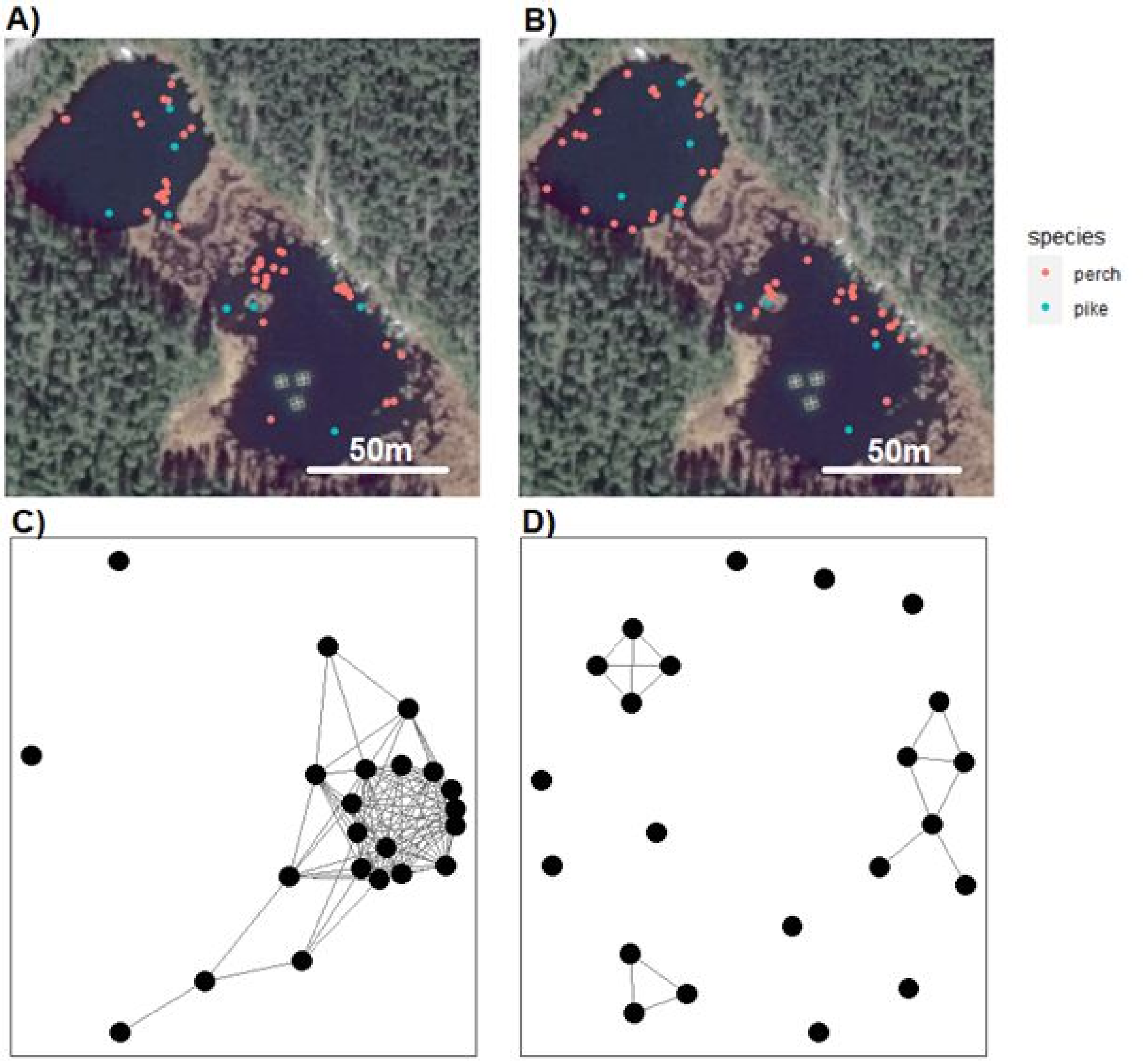

Fig. S2. **Spatial positioning converted into lake shoaling behavior**. Satellite image showing the positioning of tracked perch (red dots) and pike (blue dots) at A) one hour before dusk (mean density = ~0.3) and B) one hour after dusk (mean density = ~0.1). The data for the same hours expressed through our network analysis of perch shoaling behavior shown for the Control Lake at C) dusk and D) dawn. There, each fish (black circle) is visualized along with its associations (black lines) to other fish in the lake. The absence of lines indicates no social interaction.

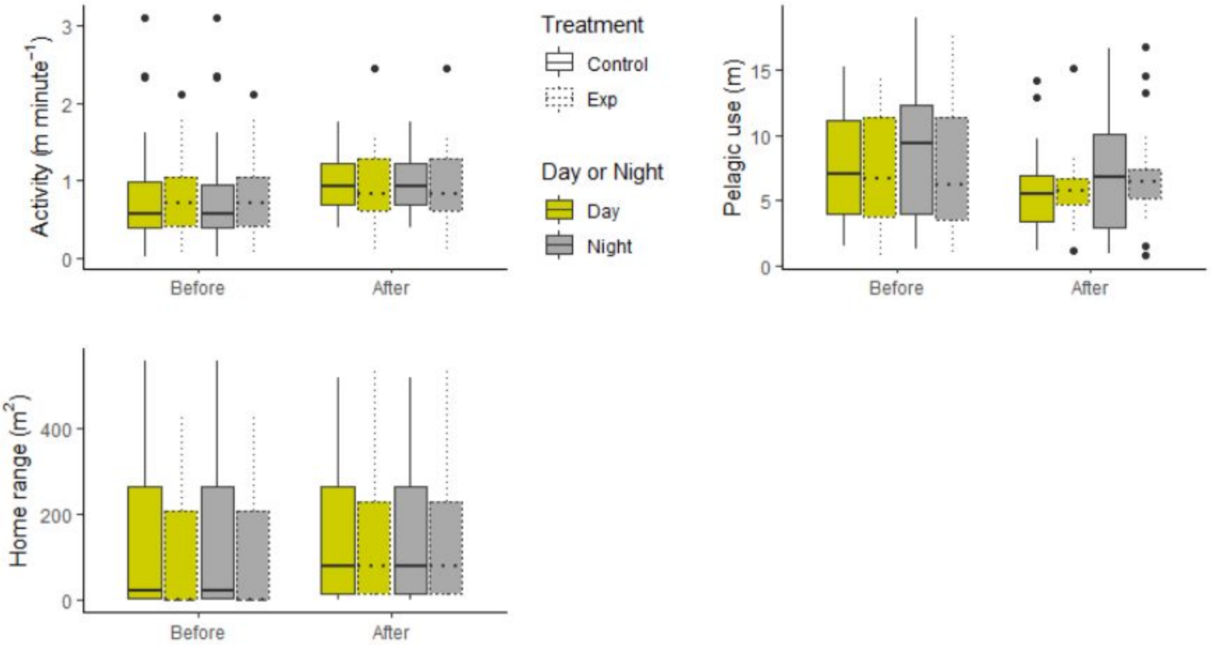

Fig. S3. **Oxazepam treatment effect on pike behavior.** Boxplots displaying the effect of oxazepam treatment on the four measured behavioral traits. Solid boxes are measurements in the Control Lake and dotted boxes in the Exposed Lake treated with oxazepam. Yellow boxes represent daytime measurements and grey boxes nighttime measurements.

Table S1. **Oxazepam measurements** during the study. Measurements were taken at every meter depth at 9 spots in the Exposed Lake. Units are in µg l^{-1}. Cntr are control measurements at two sites in the Control Lake.

Site	Depth (m)	2016-09-20	2016-09-23	2016-09-27	2016-09-30
1	Surface	15.6	N/A	9.0	13.1
1	1	10.6	10.2	14.5	13.6
1	2	13.3	11.2	12.7	10.2
1	3	14.4	9.1	16.4	8.9
2	Surface	39.7	9.0	14.0	12.1
2	1	39.4	10.2	16.5	12.8
2	2	47.7	6.4	13.9	13.1
2	3	19.2	10.2	14.5	11.8
2	4	32.9	12.0	14.4	11.1
3	Surface	27.0	12.6	11.8	11.3
3	1	25.6	9.8	13.1	13.5
3	2	21.3	12.4	15.6	14.8
3	3	32.0	12.1	15.8	12.7
3	4	23.7	11.3	12.5	11.1
4	Surface	30.6	8.6	15.5	11.6
4	1	31.7	11.5	12.2	10.2
4	2	27.6	9.4	15.9	11.3
4	3	38.0	10.6	12.7	11.7
5	Surface	14.4	10.9	11.5	11.9
5	1	16.1	9.4	13.8	9.2
5	2	16.7	9.7	13.2	10.9
5	3	11.7	10.2	15.9	10.9
5	4	10.6	9.8	11.6	11.5
5	5	12.8	10.6	12.6	12.2
6	Surface	36.8	13.6	13.9	8.0
6	1	33.2	11.9	14.7	10.7
6	2	25.1	13.0	12.1	12.6
7	Surface	18.8	12.0	20.0	13.2
7	1	33.5	11.0	15.2	12.0
8	Surface	34.2	11.3	10.7	10.2
8	1	23.0	11.2	11.9	10.1
8	2	24.4	10.4	11.0	9.6
8	3	22.5	9.2	10.9	9.6
8	4	27.1	10.2	11.2	13.1
9	Surface	12.8	10.7	10.7	11.0
9	1	13.3	9.6	10.2	11.0
9	2	18.3	11.9	9.8	11.0
9	3	18.3	9.2	11.3	9.9
Cntr 1	1	<LOQ	<LOQ	<LOQ	<LOQ
Cntr 1	2	<LOQ	<LOQ	<LOQ	<LOQ
Cntr 1	3	<LOQ	<LOQ	<LOQ	<LOQ
Cntr 2	1	<LOQ	<LOQ	<LOQ	<LOQ
Cntr 2	2	<LOQ	<LOQ	<LOQ	<LOQ
Cntr 2	3	<LOQ	<LOQ	<LOQ	<LOQ

LOQ = Limit of quantification

Table S2. **Statistical summary of best-fitting models testing for effects on the measured behavioral traits.** Summary of results from multivariate linear models (LMs) during the first part of the study (up until 2 days after oxazepam treatment). The effect of oxazepam (grey shade) on the measured behavioral traits is tested by the Time x Treatment interaction according to the BACI design. Shown are the best-fitting models selected using stepwise AIC model selection.

**Day**	**Pelagic use**			**Home range**			**Activity**			**Predator avoidance**			**Social distance**		
Predictors	*Estimates*	*CI*	*p*	*Estimates*	*CI*	*p*	*Estimates*	*CI*	*p*	*Estimates*	*CI*	*p*	*Estimates*	*CI*	*p*
(Intercept)	-50.5	-97.46 – -3.54	**0.035**	-641.32	-1867.04 – 584.39	0.3	1.56	1.12 – 2.00	**<0.001**	17.93	16.40 – 19.45	**<0.001**	11.78	8.50 – 15.07	**<0.001**
Time [Before]	136.97	35.61 – 238.33	**0.009**	31083.97	-1118.70 – 63286.65	0.06	0.37	-0.13 – 0.88	0.144	-5.23	-7.38 – -3.07	**<0.001**	-7.86	-12.05 – -3.68	**<0.001**
Treatment [Exposed]	-25.12	-45.53 – -4.71	**0.016**	-397.76	-930.47 – 134.94	0.14	0.68	0.17 – 1.18	**0.009**						
Density	441.84	109.36 – 774.32	**0.01**	5977.03	-2701.14 – 14655.20	0.17							-42.15	-61.02 – -23.28	**<0.001**
Time [Before] * Density	-749.58	-1310.74 – -188.42	**0.009**	-131988.34	-267309.04 – 3332.35	0.06							41.63	18.71 – 64.55	**0.001**
Time [Before] * Treatment [Exposed]				-10909.42	-22945.87 – 1127.03	0.08									
Observations	88			88			88			88			88		
R^2 / R^2 adjusted	0.129 / 0.087			0.389 / 0.352			0.098 / 0.077			0.213 / 0.204			0.215 / 0.187		

**Night**	**Pelagic use**			**Home range**			**Activity**			**Predator avoidance**			**Social distance**		
Predictors	*Estimates*	*CI*	*p*	*Estimates*	*CI*	*p*	*Estimates*	*CI*	*p*	*Estimates*	*CI*	*p*	*Estimates*	*CI*	*p*
(Intercept)	10.1	-1.90 – 22.11	0.098	-6179032.3	-14763765.44 – 2405700.80	0.16	4.21	2.79 – 5.63	**<0.001**	-13.64	-28.51 – 1.22	0.072	2927.3	-885.39 – 6739.99	0.13
Treatment [Exposed]	-6.14	-20.67 – 8.39	0.403	2684816.27	-1054096.08 – 6423728.61	0.16	-2.86	-4.58 – -1.14	**0.001**	31.24	12.19 – 50.30	**0.002**	-3639.58	-8793.27 – 1514.12	0.164
Density	13.07	-86.35 – 112.48	0.794	46453185.1	-17927270.10 – 110833640.19	0.16	-24.16	-35.92 – -12.40	**<0.001**	227.42	97.72 – 357.13	**0.001**	109.2	13.33 – 205.06	**0.026**
Treatment [Exposed] * Density	109.64	-33.22 – 252.50	0.131	3785.33	-165.60 – 7736.26	0.06	22.71	5.81 – 39.61	**0.009**	-243.6	-426.67 – -60.52	**0.01**	-133.09	-245.62 – -20.56	**0.021**
Time [Before]				73596.86	-27037.88 – 174231.60	0.15				-5.08	-7.80 – -2.36	**<0.001**	-240.49	-503.44 – 22.45	0.072
Temperature				516984.18	-201219.25 – 1235187.61	0.16							-245.43	-564.40 – 73.55	0.13
Time [Before] * Treatment [Exposed]				-31898.75	-75829.48 – 12031.97	0.15							137.6	-11.07 – 286.28	0.069
Time [Before] * Density				-552004.99	-1304734.81 – 200724.83	0.15							1511.19	-150.98 – 3173.35	0.074
Treatment [Exposed] * Temperature				-224657.57	-537456.64 – 88141.50	0.16							305.47	-125.70 – 736.63	0.162
Density * Temperature				-3886495.7	-9272598.72 – 1499607.25	0.16									
Observations	88			88			88			88			88		
R^2 / R^2 adjusted	0.099 / 0.067			0.434 / 0.368			0.170 / 0.140			0.217 / 0.180			0.182 / 0.099		
